# Pharmacophore Modelling-Based Drug Repurposing Approaches for SARS-CoV-2 Therapeutics

**DOI:** 10.3389/fchem.2021.636362

**Published:** 2021-05-10

**Authors:** Shailima Rampogu, Keun Woo Lee

**Affiliations:** Department of Bio and Medical Big Data (BK21 Four Program), Research Institute of Natural Science (RINS), Gyeongsang National University (GNU), Jinju, South Korea

**Keywords:** SARS-CoV-2, novel coronavirus, COVID-19, drug repurposing, pharmacophore modelling

## Abstract

The recent outbreak of severe acute respiratory syndrome coronavirus 2 (SARS-CoV-2) has caused a devastating effect globally with no effective treatment. The swift strategy to find effective treatment against coronavirus disease 2019 (COVID-19) is to repurpose the approved drugs. In this pursuit, an exhaustive computational method has been used on the DrugBank compounds targeting nsp16/nsp10 complex (PDB code: 6W4H). A structure-based pharmacophore model was generated, and the selected model was escalated to screen DrugBank database, resulting in three compounds. These compounds were subjected to molecular docking studies at the protein-binding pocket employing the CDOCKER module available with the Discovery Studio v18. In order to discover potential candidate compounds, the co-crystallized compound S-adenosyl methionine (SAM) was used as the reference compound. Additionally, the compounds remdesivir and hydroxycholoroquine were employed for comparative docking. The results have shown that the three compounds have demonstrated a higher dock score than the reference compounds and were upgraded to molecular dynamics simulation (MDS) studies. The MDS results demonstrated that the three compounds, framycetin, kanamycin, and tobramycin, are promising candidate compounds. They have represented a stable binding mode at the targets binding pocket with an average protein backbone root mean square deviation below 0.3 nm. Additionally, they have prompted the hydrogen bonds during the entire simulations, inferring that the compounds have occupied the active site firmly. Taken together, our findings propose framycetin, kanamycin, and tobramycin as potent putative inhibitors for COVID-19 therapeutics.

## Introduction

Coronaviruses are the responsible agents that cause respiratory tract infections in humans (Rothan and Byrareddy, 2020). The recent outbreak has caused a serious challenge in finding effective therapeutics (Cao et al., 2020). SARS-CoV-2 has originated from Coronaviridae family and is known as novel coronavirus (2019-nCoV) (Pal et al., 2020; Zhou et al., 2020). Being the largest group of viruses, Coronaviruses (CoVs) has four families, of which the *Coronavirinae* a subfamily of *Coronaviridae* family can be divided into α, β, γ, and δ (Woo et al., 2010; Fehr and Perlman, 2015). These viruses have a club-shaped protrusion on their surface, resembling a “crown” referring to “corona” in *Latin* (Li, 2016; Chorba, 2020). COVID-19 is the seventh type of human infecting coronavirus (Andersen et al., 2020).

The SARS-CoV-2 genome is made up of nonstructural polyprotein, open reading frame (ORF)1a/b and about 30 kb in size. It is further cleaved proteolytically to generate 15/16 proteins, 4 structural and 5 accessory proteins (Chan et al., 2020; Sinha et al., 2020; Wu et al., 2020). When the spike protein of SARS-CoV-2 (COVID-19) interacts with ACE-2, the infection initiates aided by the enzyme TMPRSS2 protease (Hoffmann et al., 2020; Mousavizadeh and Ghasemi, 2020). Once the initiation of the infection happens, ORF1a and ORF1ab are translated and subsequently cleaved proteolytically to form functional proteins and are majorly involved in viral replication (Mousavizadeh and Ghasemi, 2020; Krichel et al., (2020)).

In order to escape the degradation process and to secure efficient translation and additionally to escape from the host innate immune system recognition (Encinar and Menendez, 2020), the RNA molecule of SARS-CoV-2 (COVID-19) is capped at their 5′ end. One of the nonstructural proteins, nsp16, encodes the 2′-O-methyltransferase (2′-O-MTase) and executes the RNA cap modification, which requires nsp10 for activation. This unique feature of SARS-CoV-2 is not discovered in host cell or other viruses. The binding of nsp10 to nsp16 happens through a ∼930 Å^2^ activation surface in nsp10 (Decroly et al., 2011) correspondingly, favoring the nsp16 interaction to capped RNA substrate and methyl donor S-adenosyl-l-methionine (SAM), thus stabilizing the SAM-binding pocket and elongating the capped RNA-binding groove (Encinar and Menendez, 2020). A recent finding has documented three binding sites for the nsp16/nsp10 complex, the SAM-binding site, the interface between the nsp16-nsp10, and the RNA-binding groove (Encinar and Menendez, 2020). In the current investigation, we have targeted the SAM-binding site for exploiting new leads to combat SARS-CoV-2, adapting several computational approaches.

With no specific medication till date, it is essential to develop and/or design new therapeutics to counter this pandemic. A host of drugs are under investigation, and the search for new compounds is underway. Here, we targeted the nonstructural protein, the nsp16/nsp10 complex, to identify an effective prospective drug employing the *drug repurposing* approach using several computational studies. Additionally, the use of the already-known antiviral drugs offers several advantages due its known safety profiles (Das et al., 2020).

## Materials and Methods

### Selection of the Target Structure

The protein for the present study is the nsp16/nsp10 complexed with the co-crystalized ligand SAM bearing the PDB code 6W4H (Rosas-Lemus et al., 2020). The protein was prepared by removing the water molecules and the heteroatoms. Subsequently, the protein was minimized enabling the “*Minimize and Refine Protein*” protocol available with the Discovery Studio v18 (DS). The chain A of the protein was used, and the active site residues were marked around the SAM ligand at 12 Å radius.

### Pharmacophore Generation and Virtual Screening Process

The receptor ligand pharmacophore model was generated exploiting the key interacting features present between the target and SAM. Correspondingly, the *Receptor-Ligand Pharmacophore Generation* module was used retaining all the other parameters as default (Meslamani et al., 2012). The best pharmacophore model was chosen based upon the selectivity score and the key residue interactions and was upgraded to screen the small molecules.

### Preparation of the Small Molecule Dataset

The FDA-approved small molecule dataset was downloaded from the DrugBank (Wishart et al., 2018) in the .sdf format and was subsequently imported onto the DS. The small molecules were initially checked for the presence of any duplicates and were thereafter minimized employing the “*Full Minimization*” protocol accessible with the DS.

### Binding Affinity Studies

Molecular docking was conducted to evaluate the binding affinities between the target protein and the small molecules. Herein, the CDOCKER (Wu et al., 2003) program available with the DS was employed. Each ligand was allowed to generate 50 conformations, and the best pose was selected based upon the highest dock score (-CDOCKER interaction energy) from the largest cluster and the key residue interactions (Rampogu et al., 2018). The compound SAM was used as the reference compound, while remdesivir and hydroxychloroquine were used for comparative docking.

### Molecular Dynamics Simulation Studies

Molecular dynamics simulation (MDS) studies were undertaken to envisage on the behavior of the ligand at the binding pocket of the protein. The protein parameters were generated from the CHARMM 27 all-atom force field, and the ligand topologies were garnered from SwissParam (Zoete et al., 2011) employing the GROMACS v 2016.6 (Van Der Spoel et al., 2005). The systems were solvated in dodecahedron water box consisting of TIP3P water model and, subsequently, the counter ions were added. The systems were subsequently minimized followed by coupling of the protein and the ligand. This was proceeded by double equilibration method using the conserved number of particles (N), volume (V) and temperature (T) (NVT) and the constant number of particles (N), pressure (P), and temperature (T) (NPT) for 1 ns each. The NPT ensembles were escalated to MDS for 50 ns. All the analyses were carried out using visual molecular dynamics (VMD) (Humphrey et al., 1996) and DS. The results were evaluated according to the root mean square deviation (RMSD), root mean square fluctuations (RMSFs), radius of gyration (Rg), potential energy, number of hydrogen bonds, distance between the hydrogen bond interacting residues and ligand atoms, interaction energy, and the mode of ligand binding.

## Results

### Pharmacophore Generation

**FIGURE 1:**
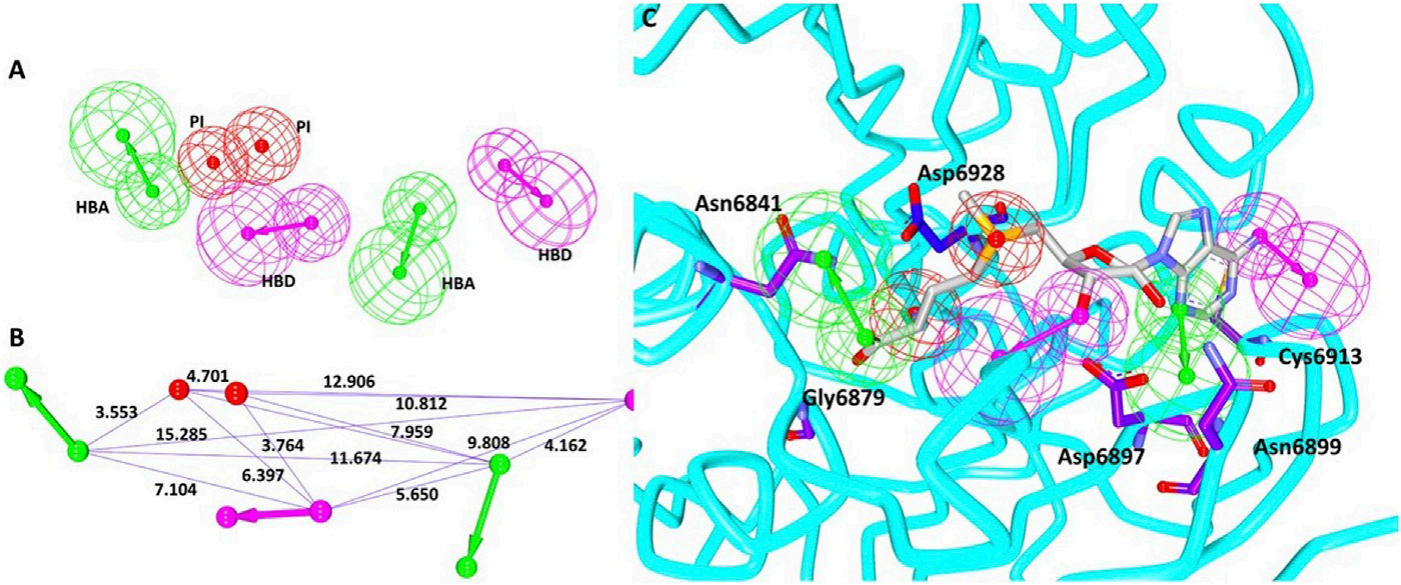
Pharmacophore model generation. **(A)** Pharmacophore features. **(B)** Geometry of the model. **(C)** Model complementary to the residues and features.

Utilizing the interactions between the co-crystallized ligand SAM and the nsp16 target protein, the structure-based pharmacophore model was generated. The results have generated 10 pharmacophore models with the same features and selectivity score as shown in Table 1. When the receptor–ligand generation protocol was initiated, a total of 37 features in ligand were observed. However, 10 features have matched the receptor–ligand interactions: AAAAAADDPP [where A refers to hydrogen bond acceptor (HBA), D refers to hydrogen bond donor (HBD), and P indicates positive ionizable (PI)], out of which six features were incorporated in each model. For the scoring method, the *Rules* was used, which is an internal scoring method. This scoring function is based on a genetic function approximation (GFA model), which is a function of the feature set in the pharmacophore model and the feature–feature distances of different types of features. In order to select the ideal model, the pharmacophore that covers the entire structure of the SAM along with the key residue interactions was selected. Accordingly, model 3 was selected with the following features: 2 hydrogen bond acceptors (HBAs), 2 hydrogen bond donors (HBDs), and 2 positive ionizables (PIs), as shown in Figure 1A with its geometry in Figure 1B. These features were complementary to the key residues such as Asn6841, Asp6897, Asn6899, Cys6913, and Asp6928, respectively, as illustrated in Figure 1C. We then evaluated if the model could retrieve potential inhibitors. Since there are not many compounds specific to nsp16/nsp10 complex, the pan-MTase inhibitor sinefungin was used. This compound is bound to SARS-CoV-2 nsp10-nsp16 methyltransferase complex bearing the PDB code 6YZ1 (Krafcikova et al., 2020). Upon enabling the *Ligand Pharmacophore Mapping* tool embedded with the DS, it was revealed that the compound has mapped perfectly with the model, Supplementary Figure S1 suggesting that the model could be upgraded for retrieving new potential inhibitors.

**TABLE 1:** Generation of different pharmacophore models and their characterization.

Pharmacophore model	Number of features	Feature set^a^	Selectivity score
Model 1	6	HBA, HBA, HBD, HBD, PI, PI	14.834
Model 2	6	HBA, HBA, HBD, HBD, PI, PI	14.834
Model 3	6	HBA, HBA, HBD, HBD, PI, PI	14.834
Model 4	6	HBA, HBA, HBD, HBD, PI, PI	14.834
Model 5	6	HBA, HBA, HBD, HBD, PI, PI	14.834
Model 6	6	HBA, HBA, HBD, HBD, PI, PI	14.834
Model 7	6	HBA, HBA, HBD, HBD, PI, PI	14.834
Model 8	6	HBA, HBA, HBD, HBD, PI, PI	14.834
Model 9	6	HBA, HBA, HBD, HBD, PI, PI	14.834
Model 10	6	HBA, HBA, HBD, HBD, PI, PI	14.834

^a^ HBA refers to hydrogen bond acceptor, HBD refers to hydrogen bond donor, and PI indicates positive ionizable.

### Pharmacophore Mapping to Discover New Indications for FDA-Approved Small Molecules

The prepared FDA-approved drugs were mapped with the pharmacophore model enabling the *Ligand Pharmacophore Mapping* tool, which resulted in three compounds, as shown in Supplementary Figure S2, inferring that these compounds possess the important features vital for inhibition. The resultant compounds were upgraded to binding affinity studies employing the CDOCKER program available with DS.

### Binding Affinity Studies

The molecular docking was undertaken to predict the binding modes of the small molecules and further to estimate their binding affinities (Rampogu et al., 2020). Upon subjecting the obtained compounds to molecular docking mechanism along with the reference compound, three compounds were found to generate a higher to comparable dock score than the reference compound, as displayed in Table 2. Therefore, these compounds were upgraded to MDS studies to evaluate their stabilities.

**TABLE 2:** Binding affinity scores according to the CDOCKER.

Compound name	DrugBank ID	-CDOCKER interaction energy (kcal/mol)
Framycetin	DB00452	68.80
Kanamycin	DB01172	62.18
Tobramycin	DB00684	58.40
S-Adenosylmethionine	Reference compound	64.26
Remdesivir		57.16
Hydroxychloroquine		49.13

### Molecular Dynamics Simulation Studies

Molecular dynamics simulation study was carried out to understand the behavior of small molecules at the binding pocket of the target. The results were delineated based upon the root mean square deviation (RMSD), radius of gyration (Rg), potential energy, root mean square fluctuation (RMSF), mode of ligand binding, number of hydrogen bond analysis, and the interaction energy.

### MDS Inferred Stability Analysis

#### Root Mean Square Deviation

**FIGURE 2:**
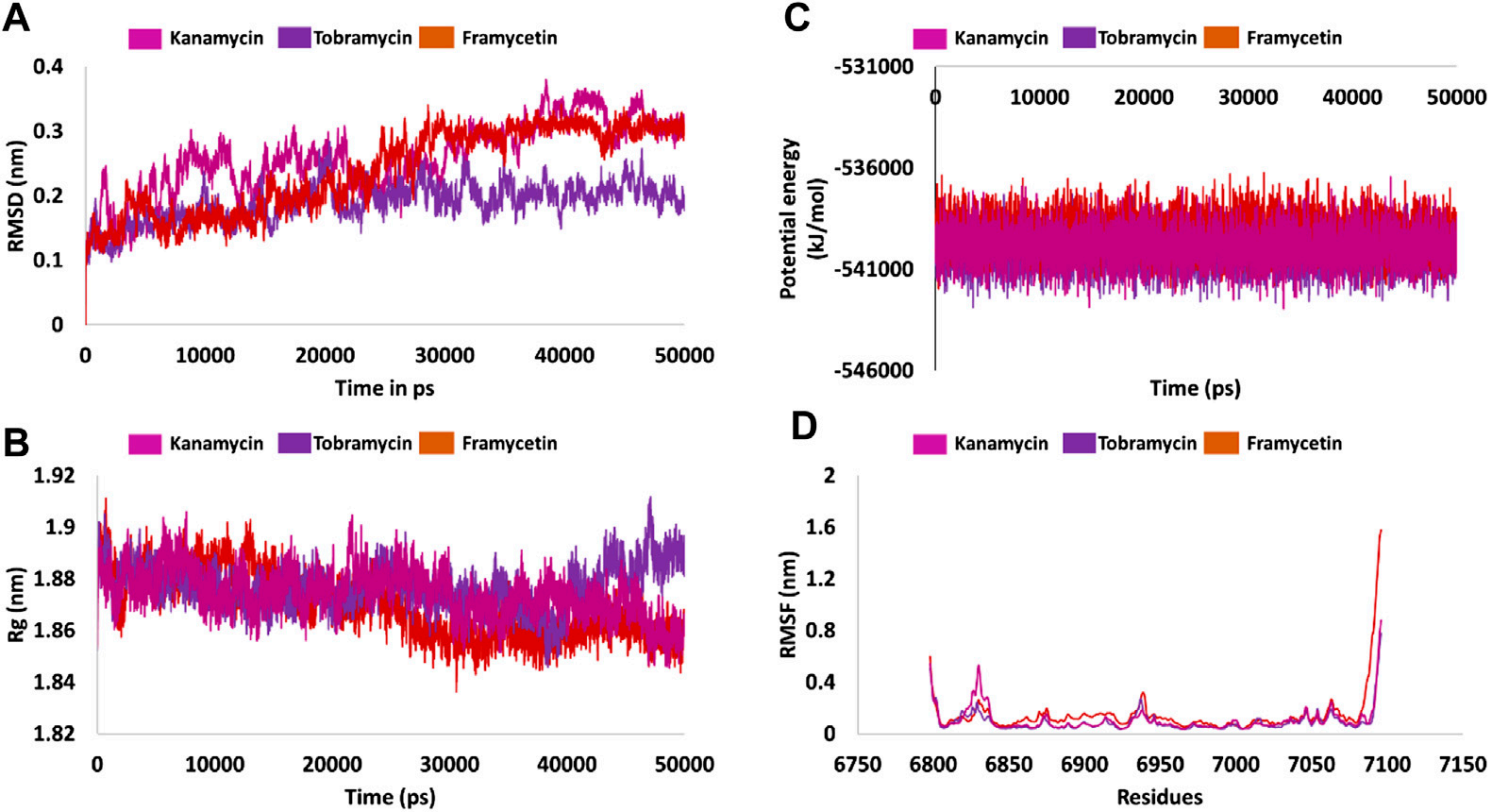
Molecular dynamics simulation findings. **(A)** Stability analysis as inferred by RMSD. **(B)** Compactness of the protein from Rg. **(C)** Potential energy during the entire simulation. **(D)** Fluctuations rendered by the RMSF plots.

Protein stability with respect to its conformation can be measured *via* the deviations generated, if any, during the simulation period, logically inferring that the smaller the deviation the greater the stability of the protein (Aier et al., 2016). Correspondingly, the RMSD plots were plotted for the protein backbone atoms to examine if there are any variations in the plot. The RMSD plot was found to be stable below 0.23 nm for framycetin, an average of 0.18 nm for tobramycin, and an average of 0.25 nm for kanamycin. These findings elucidate that the system is stable, demonstrating no major aberrations, as described in Figure 2A.

#### Radius of Gyration

Radius of gyration serves as an indicator for protein structure compactness, further stating that the three-state folding of the proteins is relatively more compact than the proteins of the same size but in two-state folding (Lobanov et al., 2008). In the current study, we investigated the compactness of the protein (backbone) during the whole simulation. It was noted that the protein was highly stable ranging between 1.86 and 1.90 nm. Interestingly, the Rg of the three systems was observed to drop at 25 ns thereafter being stable. However, in case of tobramycin, the Rg has raised marginally at 40 ns and remained stable thereafter. Overall, the three systems have revealed a greater compactness as shown in Figure 2B.

#### Potential Energy

Another parameter, that is, the potential energy, was also calculated. The three systems have displayed a stable potential energy between −542000 and −537000 kJ/mol. The average potential energy was calculated as −539864 kJ/mol for kanamycin, −539133 kJ/mol for framycetin, and −540066 kJ/mol for tobramycin. These results additionally strengthen the argument that the systems were stable with no major variations, as shown in Figure 2C.

#### Root Mean Square Fluctuation

Root mean square fluctuation (RMSF) is defined as a measure of residue-specific flexibility (Dong et al., 2018). Here, the RMSF was conducted on the protein backbone of the three systems. It was observed that there were no fluctuations noticed in the backbone residues, as shown in Figure 2D. The binding pocket is made up of the residues Asn6841, Tyr6845, Gly6879, Gly6871, Pro6878, Gly6879, Asp6897, Leu6898, Asn6899, Asp6912, Cys6913, Asp6928, Met6929, and Tyr6930. Interestingly, no significant fluctuations were noticed with these residues as described in Supplementary Figure S3A. The residues between 6,820–6,830 and 6,932–6,942 were observed to demonstrate a marginal surge in the RMSF plots. However, these residues do not belong to the active site as shown in Figure 2D and Supplementary Figure S3B. The putative inhibitors were accommodated at the binding pocket by interacting with the key residues. The detailed computational analysis has unraveled their binding potential at the atomistic level. Furthermore, mechanistically, we speculate that binding with these key residues could illuminate the credibility of the compounds as new inhibitors.

### Binding Mode Analysis

**FIGURE 3:**
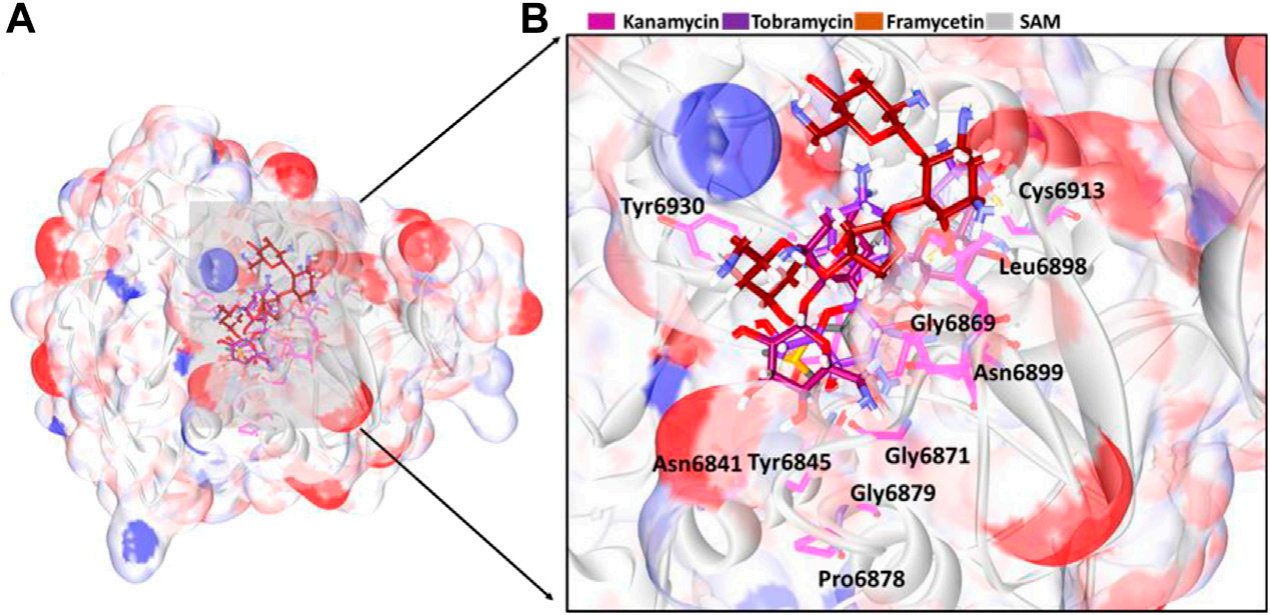
Binding mode analysis of the compounds. **(A)** Accommodation of ligands at the binding pocket and **(B)** its enlarged view surrounded by the binding pocket residues.

**FIGURE 4:**
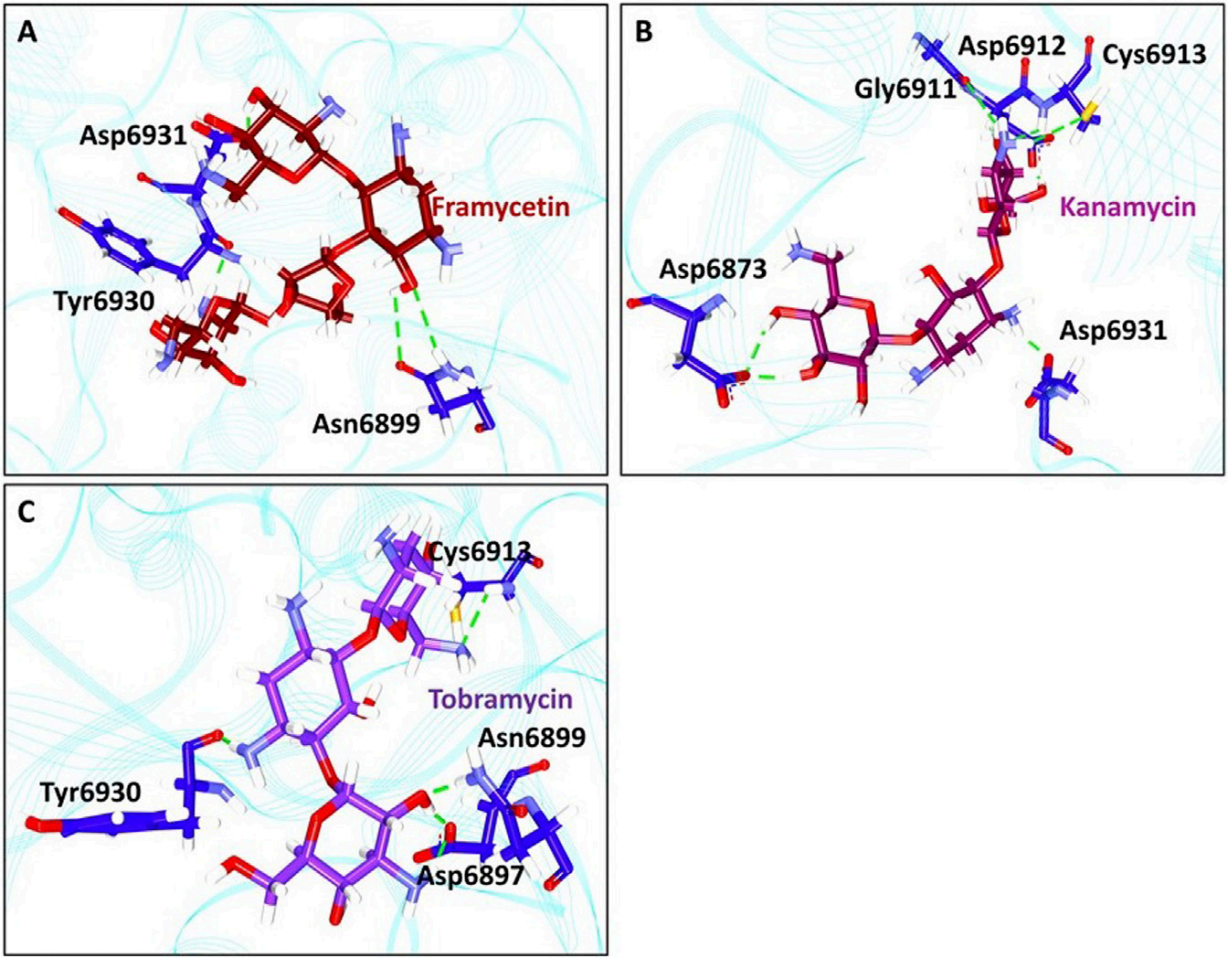
Intermolecular hydrogen bond interactions. **(A)** Molecular interactions between framycetin and the key residues. **(B)** Hydrogen bond interactions between kanamycin and the protein residues. **(C)** Intermolecular hydrogen bond interactions between tobramycin and the protein residues.

From the stable RMSD, the last 5 ns structure was extracted and was superimposed against the X-ray crystal structure. The results have revealed that the compounds have occupied the same binding pocket as that of the co-crystallized compound SAM, as shown in Figure 3. The intermolecular interactions have shown that the compound framycetin has formed four hydrogen bond interactions with residues Asn6899, Tyr6930, and Asp6931. The residue Asn6899 has generated two hydrogen bonds as illustrated in Figure 4A. Additionally, the residues, Leu6898, Met6929, and Lys6933 have formed the van der Waals interactions firmly holding the compound at the binding pocket as illustrated in Supplementary Figure S5.

The compound kanamycin has occupied the similar binding pocket as that of the co-crystallized compound SAM, as shown in Figure 3. The intermolecular interactions have shown that the compound has formed hydrogen bond interactions with residues Asp6873, Gly6911, Asp6912, Cys6913, and Asp6931, respectively. The residues Asp6873 and Cys6913 have generated two hydrogen bonds as illustrated in Figure 4B. Additionally, the compounds Phe6868, Ser6896, Met6929, Gly6869, Asp6897, Ser6872, Gly6871, Pro6932, Lys6933, and Leu6898 have formed the van der Waals interactions firmly holding the compound at the binding pocket, as illustrated in Supplementary Figure S5.

The compound tobramycin has occupied the binding pocket in the same fashion as that of the co-crystallized compound SAM, as shown in Figure 3. The intermolecular interactions have shown that the compound has formed hydrogen bond with residues Asn6899, Asp6897, Cys6913, and Tyr6930, respectively. The residue Asp6897 has generated two hydrogen bonds as illustrated in Figure 4C. Additionally, the compounds Ala6914, Asp6912, Tyr6950, Phe6947, Lys6933, Asp6931, Pro6932, Asp6873, Ser6872, Gly6871, and Gly6911 have formed the van der Waals interactions accommodating the compound at the binding pocket, as illustrated in Supplementary Figure S5.

### Hydrogen Bond Interactions and Distance Between the Interacting Residues and Atoms

**FIGURE 5:**
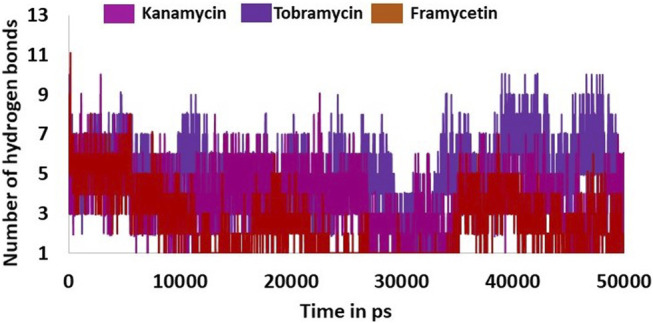
Hydrogen bond interactions between the protein and ligand during the whole simulations.

**FIGURE 6:**
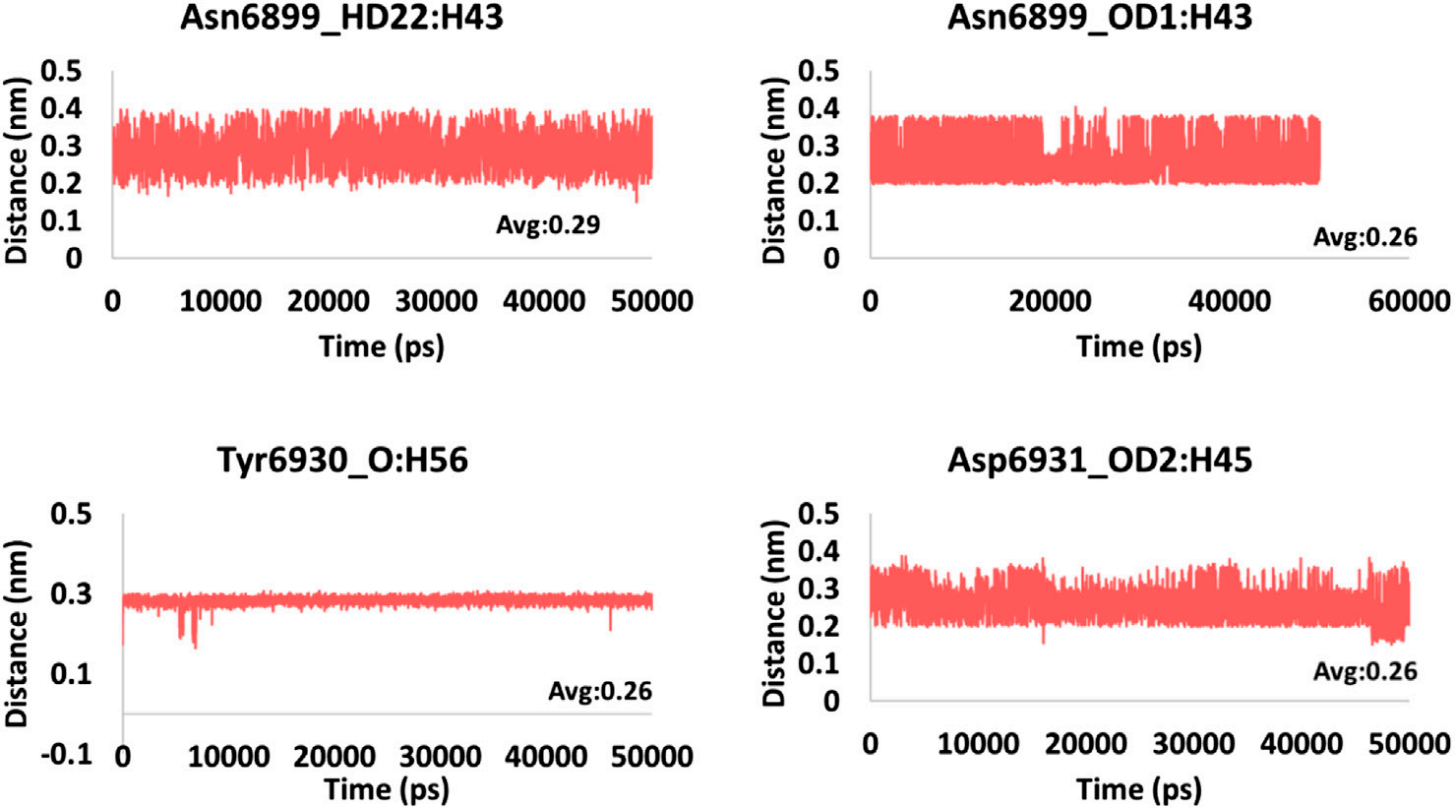
Hydrogen bond distance of the interacting residues (atoms) and the ligand atoms of framycetin.

**FIGURE 7:**
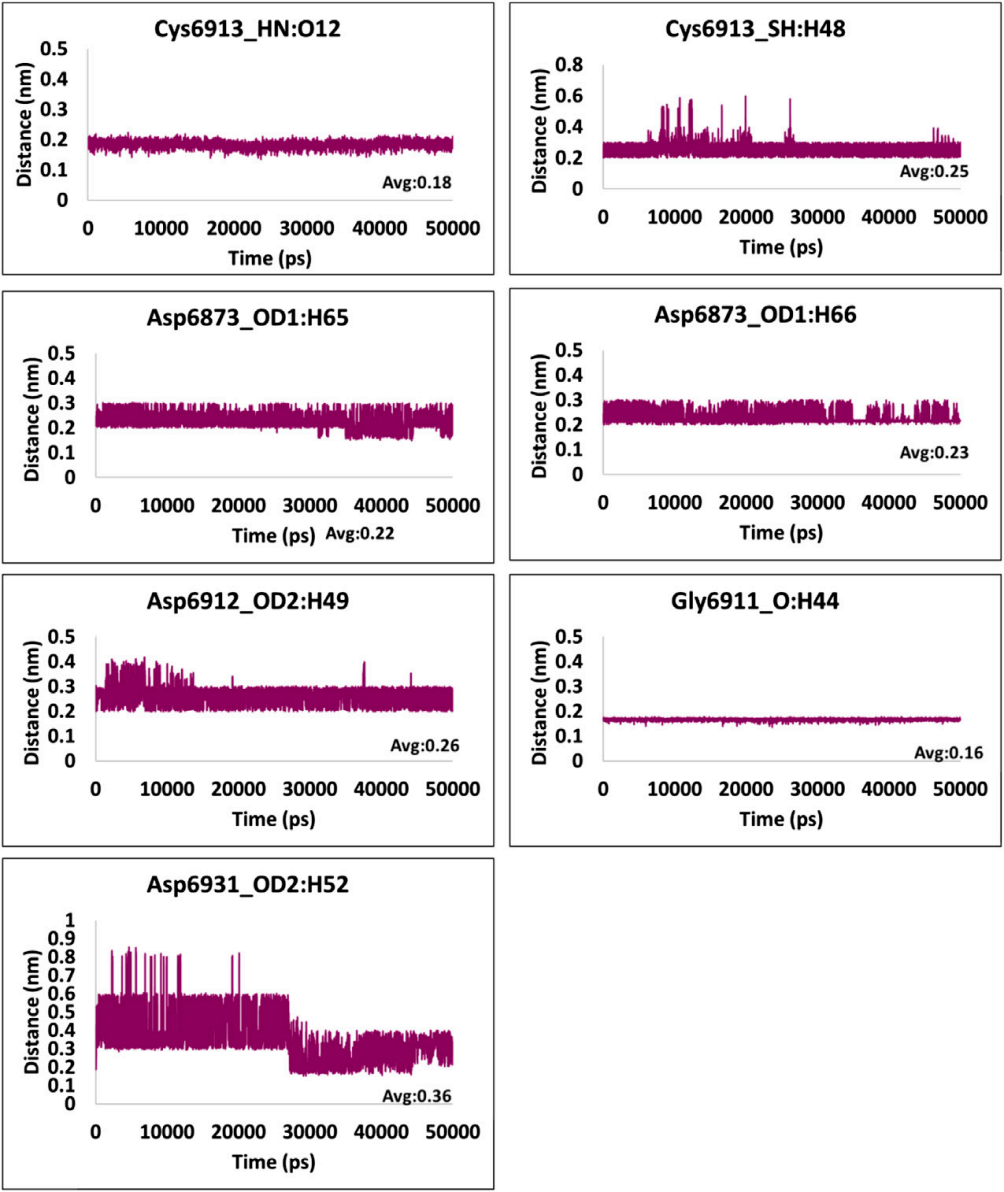
Hydrogen bond distance of the interacting residues (atoms) and the ligand atoms of kanamycin.

**FIGURE 8:**
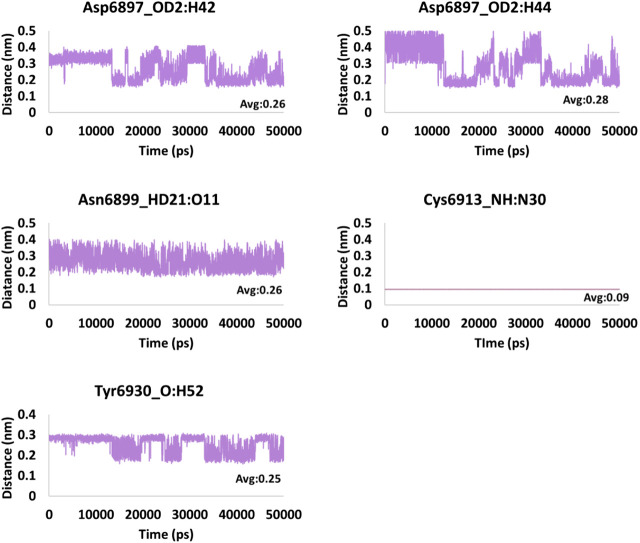
Hydrogen bond distance of the interacting residues (atoms) and the ligand atoms of tobramycin.

The hydrogen bond interactions were monitored during the 50 ns simulation run. It was noted that the hydrogen bonds were formed during the entire simulations, stating that the compounds were present within the active site of the protein. The average hydrogen bonds were computed to be 2.5, 3.8, and 4.8 for framycetin, kanamycin, and tobramycin, respectively, as displayed in Figure 5. Additionally, we meticulously investigated the distance between the interacting residues that are making the hydrogen bonds and the ligand atoms. The compound framycetin forms four hydrogen bonds noticed to be interacting throughout the simulation run with an acceptable average distance below 0.3 nm, as shown in Figure 6. The seven hydrogen bonds demonstrated by the compound kanamycin have displayed an acceptable average distance of 0.3 nm. A distance beyond 0.3 nm was noticed with Asp6912_OD2:H49, Cys6913_SH:H48 during the initial simulation run, thereafter demonstrating an acceptable bond length. The interaction, Asp6931_OD2:H52 showed longer distance that ranged from 0.3 nm to 0.9 nm until 30 ns, after which the distance has settled at an acceptable bond length projecting an average distance of 0.36 nm as described in Figure 7. The compound tobramycin has shown relatively high degree of variation in the distance measure, while maintaining the average distance below 0.3 nm. The interactions with residue Cys6913 and Asn6899 were highly rigid, illuminating their strong affinity toward the ligand, as described in Figure 8. Notably, the residue Asp has displayed high degree of variation, whilst maintaining the distance below 0.3 nm, as described in Figure 8.

### Interaction Energy Analysis

**FIGURE 9:**
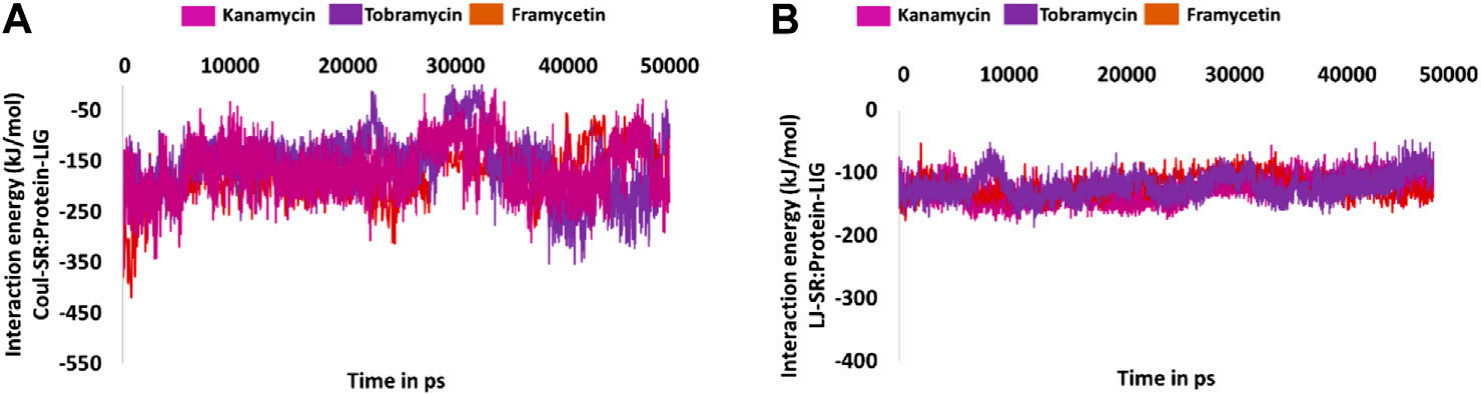
Molecular dynamics simulation–guided interaction energy during the whole simulation. **(A)** Interaction energy from Coul-SR:Protein-lig and **(B)** interaction energy from LJ-SR:Protein-lig.

Furthermore, to quantify the strength of the interaction between the protein and the small molecule, the interaction energies were computed and read according to the terms Coul-SR:protein–lig and LJ-SR:protein–lig. For framycetin, the Coul-SR: protein–lig interaction energy existed between −400 and −100 kJ/mol with an average of −178.28 kJ/mol. The LJ-SR:protein–lig interaction energy was −200 to −50 kJ/mol with an average of −121.35 kJ/mol. For kanamycin, the Coul-SR:protein–lig interaction energy existed between −400 and −100 kJ/mol with an average of −167.46 kJ/mol. The LJ-SR:protein–lig interaction energy was −100 to −200 kJ/mol with an average of −127.43 kJ/mol. For tobramycin, the Coul-SR:protein–lig interaction energy existed between −300 and −100 kJ/mol with an average of −158.01 kJ/mol. The LJ-SR:protein–lig interaction energy was −200 to −100 kJ/mol with an average of −121.15 kJ/mol, as illustrated in Figures 9A,B.

## Discussion

COVID-19 is a global pandemic (Osier et al., 2020) that the world has been witnessing with no effective medication yet. The World Health Organization (WHO) has stated that COVID-19 is “public enemy number 1” and potentially more powerful than terrorism (Yi et al., 2020). To find promising therapeutics, in the current research, we have adapted computational drug repurposing approaches.

Drug repurposing (drug repositioning, reprofiling, or re-tasking) is a method for discovering new uses for approved or investigational drugs different from the original use, and has several advantages such as reduced risk of failure, reduced time frame for drug development, and less investment (Pushpakom et al., 2018).

A few reports exist targeting the SAM binding site. Encinar et al. identified twelve drugs that have occupied the SAM binding site with a high affinity (Encinar and Menendez, 2020). In another study, Tazikeh-Lemeski et al. reported the drugs raltegravir and maraviroc, to be effective against nsp 16 (Tazikeh-Lemeski et al., 2020). Bilal et al. discovered naphthyridine and quinoline derivatives as possible nsp16-nsp10 inhibitors (Aldahham et al., 2020). However, these computational investigations have not reported our compounds.

Herein, we have used pharmacophore-based molecular docking and molecular dynamics simulation methods to discover potential candidates for SARS-CoV-2, targeting the 6W4H. Upon performing the structural alignment using the protocol *Align Structures* of SARS-CoV (3R24) and SARS-CoV-2 (6W4H), both the sequences have shown a sequence identity of 86.8% and sequence similarity of 89.4%, as shown in Supplementary Figure S4B. Additionally, upon superimposition of the two structures, it was revealed that the SAM binding site was similar, inferring that the two structures share high similarity, as shown in Supplementary Figure S4A.

Our investigation has retrieved framycetin, kanamycin, and tobramycin as potential candidate drugs as they have demonstrated higher dock score than the reference compound. In order to understand their affinities, in case to avoid false positives and to elucidate the behavior of these compounds at the active site of the target, the MDS analysis was undertaken. The results have shown that the three compounds have projected acceptable results, whilst maintaining the key residue interactions with the target.

The antibiotic framycetin is generally employed to treat bacterial eye infections (Wishart et al., 2006; Wishart et al., 2018). The compound framycetin has formed hydrogen bonds with residues Asn6899, Tyr6930, and Asp6931. The residue Asn6899 has generated a hydrogen bond with remdesivir, while demonstrating a van der Waals interaction with hydroxychloroquine. The residues Tyr6930 and Asp6931 have prompted a van der Waals interaction with the reference compounds. The compound framycetin was also found to be a potential inhibitor against SARS-CoV-2 M^pro^ (Rampogu and Lee, 2021). Kanamycin is an aminoglycoside antibiotic and acts by binding to the bacterial 30S ribosomal subunit, leading to misread t-RNA, thereby leaving the bacterium unable to synthesize proteins vital to its growth (Wishart et al., 2006; Wishart et al., 2018). The compound kanamycin has established hydrogen bond interactions with residues Asp6873, Gly6911, Asp6912, Cys6913, and Asp6931, respectively. The residue Asp6873 has formed van der Waals interaction with remdesivir and hydroxychloroquine. The residue Gly6911 has generated a van der Waals interaction with the docked pose of SAM and the cocrystallized structure. The residue Asp6912 has generated hydrogen bond interaction with the docked pose of SAM and the co-crystallized structure. The residue Cys6913 has formed van der Waals interaction with remdesivir and π-alkyl interaction with hydroxychloroquine. Interestingly, this residue has interacted with the hydrogen bond interaction in both the docked pose of SAM and the co-crystallized structure. The residue Asp6931 has formed van der Waals interaction with remdesivir and the docked pose of SAM. The compound tobramycin is a broad-spectrum aminoglycoside, antibiotic produced by *Streptomyces tenebrarius* (Wishart et al., 2006; Wishart et al., 2018). It is effective against Gram-negative bacteria, especially the *pseudomonas* species. The compound tobramycin has formed five hydrogen bonds. The residue Asp6897 has formed hydrogen bond with tobramycin as was noticed with all the reference compounds. The residue Asn6899 has formed hydrogen bond interaction with tobramycin, remdesivir, and co-crystallized ligand, while it formed van der Waals interactions with remdesivir and the docked pose of SAM. The residue Cys6913 formed hydrogen bond interactions with tobramycin, docked pose of SAM, and the cocrystallized structure. It has generated a van der Waals interaction with remdesivir. The residue Tyr6930 represented a hydrogen bond interaction with tobramycin and van der Waals interaction with hydroxychloroquine. The residue has demonstrated a carbon–hydrogen bond with remdesivir and docked pose of SAM.

Furthermore, our investigations have shown that the three compounds have been accommodated within the binding pocket during the entire simulation run without any significant variations, as predicted by RMSF profiles in Supplementary Figure S3.

Additionally, the distance plots of the hydrogen bonds determine the strength of the bonds inferring that the compounds are held strongly at the active site of the target protein. Furthermore, they were clamped by several residues at the binding pocket locking them at the active site throughout the simulation via van der Waals interaction, as shown in Supplementary Figure S5. Since these are the key residues to bring out the biological processes as noticed with the cocrystallized ligand, we speculate that our newly identified compounds could serve as effective inhibitors. Mechanistically, interacting with these residues is essential to bring about the desirable result. These compounds have also demonstrated the pharmacophore features inferring the key features as that of the cocrystallized ligand. Taken together, we propose these compounds as potential leads targeting nsp16 protein to combat SARS-CoV-2.

## Conclusion

SARS-CoV-2 causing COVID-19 is the recent pandemic the world is fighting currently with no effective therapeutics yet. In pursuit of finding effective drugs to this disease, we have performed exhaustive pharmacophore-based drug repurposing approach to discover candidate compounds from DrugBank. Our results have retrieved three potential candidates, which have shown promising computational results. These compounds have displayed the pharmacophore features as possessed by the cocrystallized compound SAM complemented by the key residue interactions and stable MDS results. Together, we propose three compounds, framycetin, kanamycin, and tobramycin, as promising therapeutics for SARS-CoV-2 infections.

## Data Availability

The original contributions presented in the study are included in the article/Supplementary Material; further inquiries can be directed to the corresponding author.
