# Supplementary material for: Pharmacophore Modelling-Based Drug Repurposing Approaches for SARS-CoV-2 Therapeutics
Source: Front Chem. 2021 May 10;9:636362. doi: 10.3389/fchem.2021.636362 (PMC8141588; doi:10.3389/fchem.2021.636362)
Supplement: Supplementary file 1 [file Presentation1.pptx]

## Slide 1
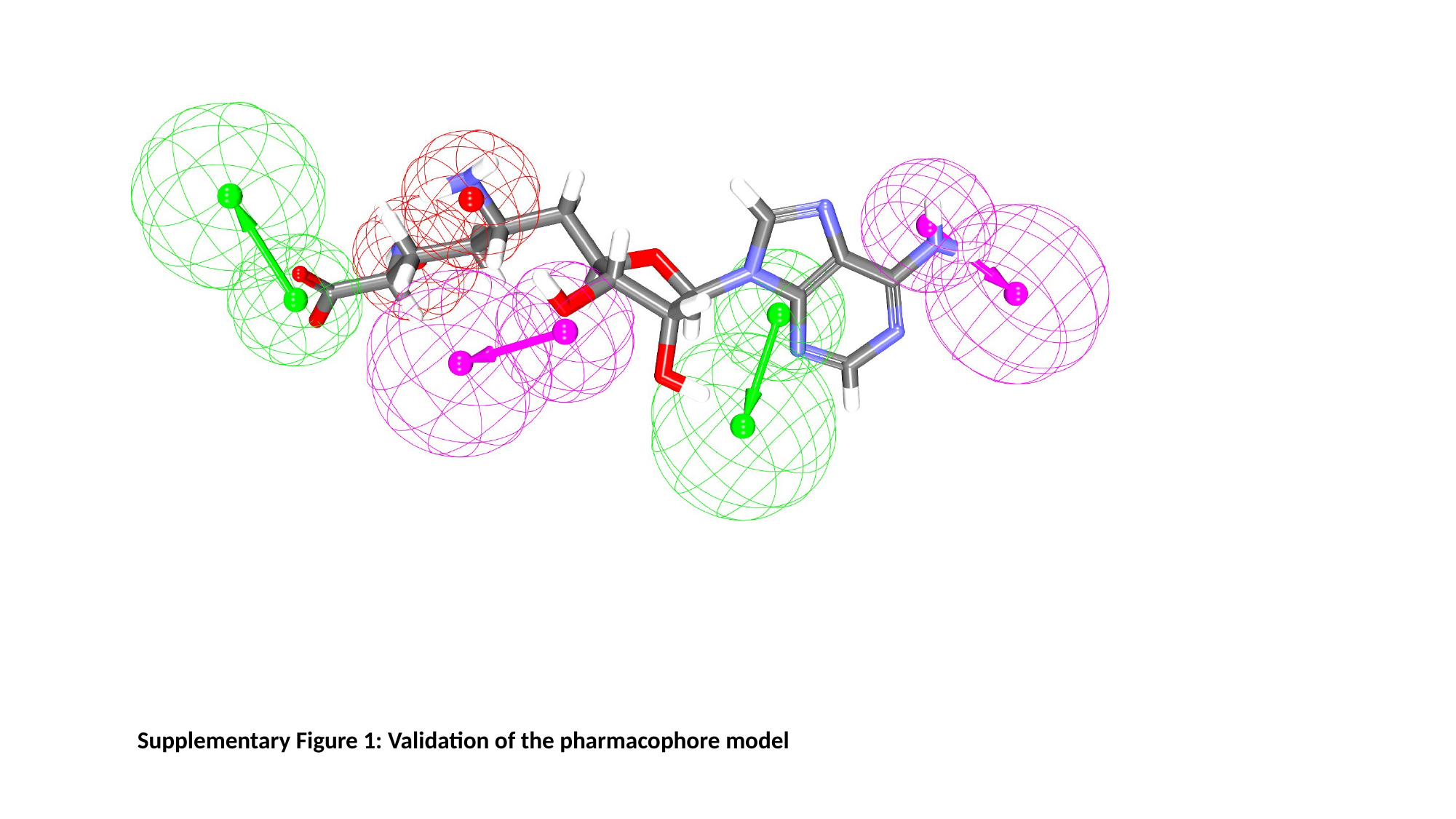

Supplementary Figure 1: Validation of the pharmacophore model

## Slide 2
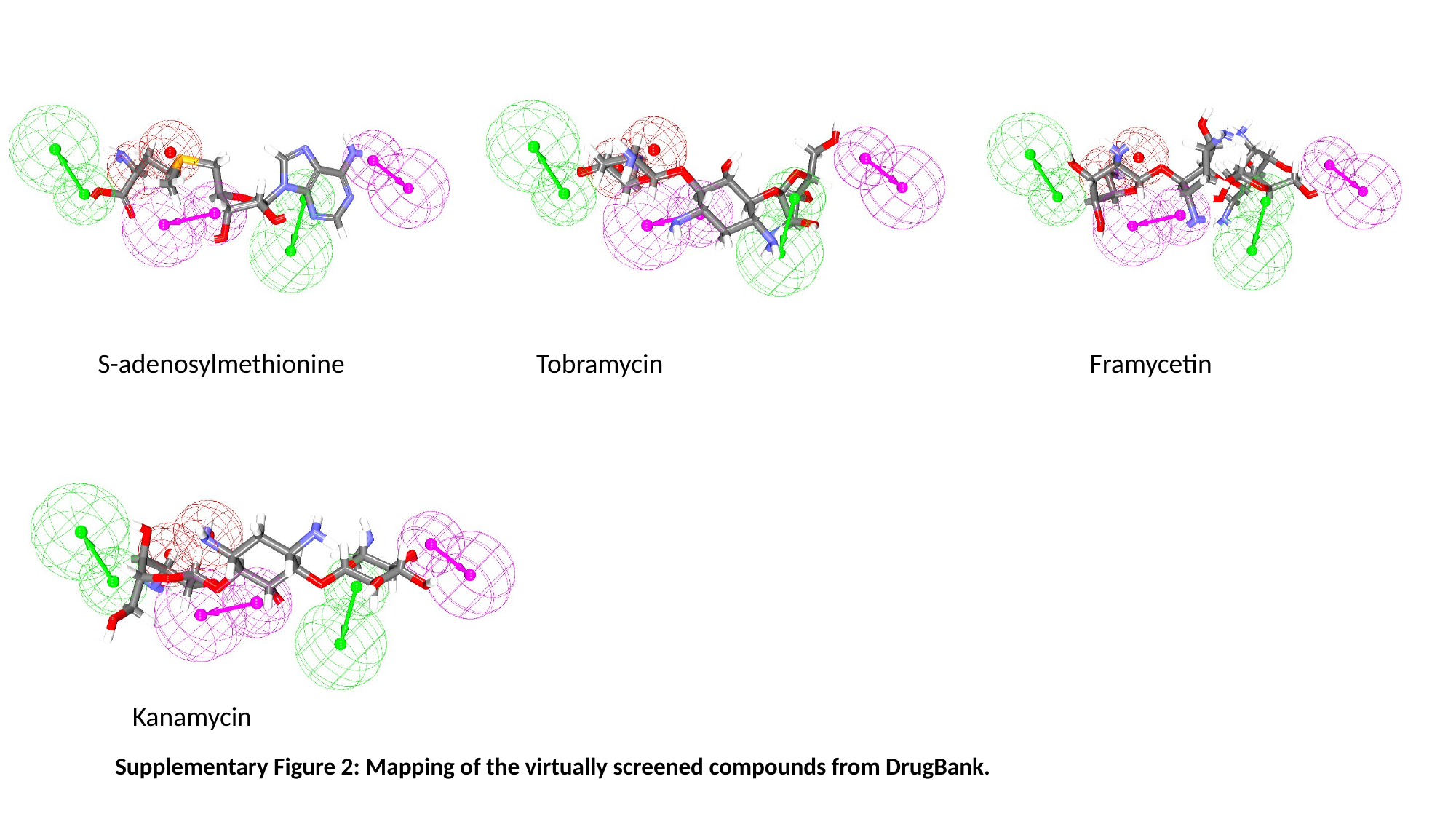

S-adenosylmethionine
Tobramycin
Framycetin
Kanamycin
Supplementary Figure 2: Mapping of the virtually screened compounds from DrugBank.

## Slide 3
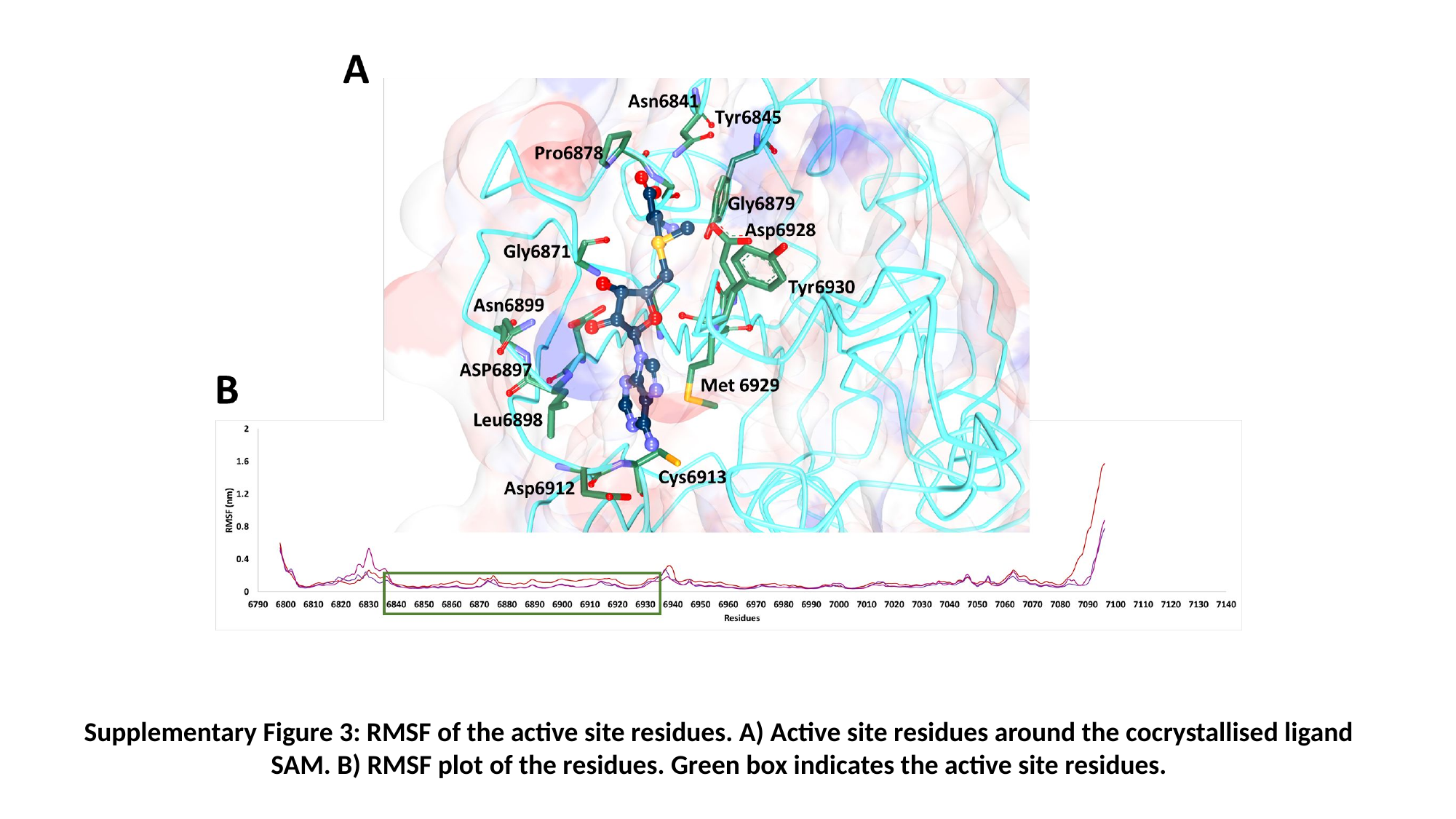

Supplementary Figure 3: RMSF of the active site residues. A) Active site residues around the cocrystallised ligand SAM. B) RMSF plot of the residues. Green box indicates the active site residues.

## Slide 4
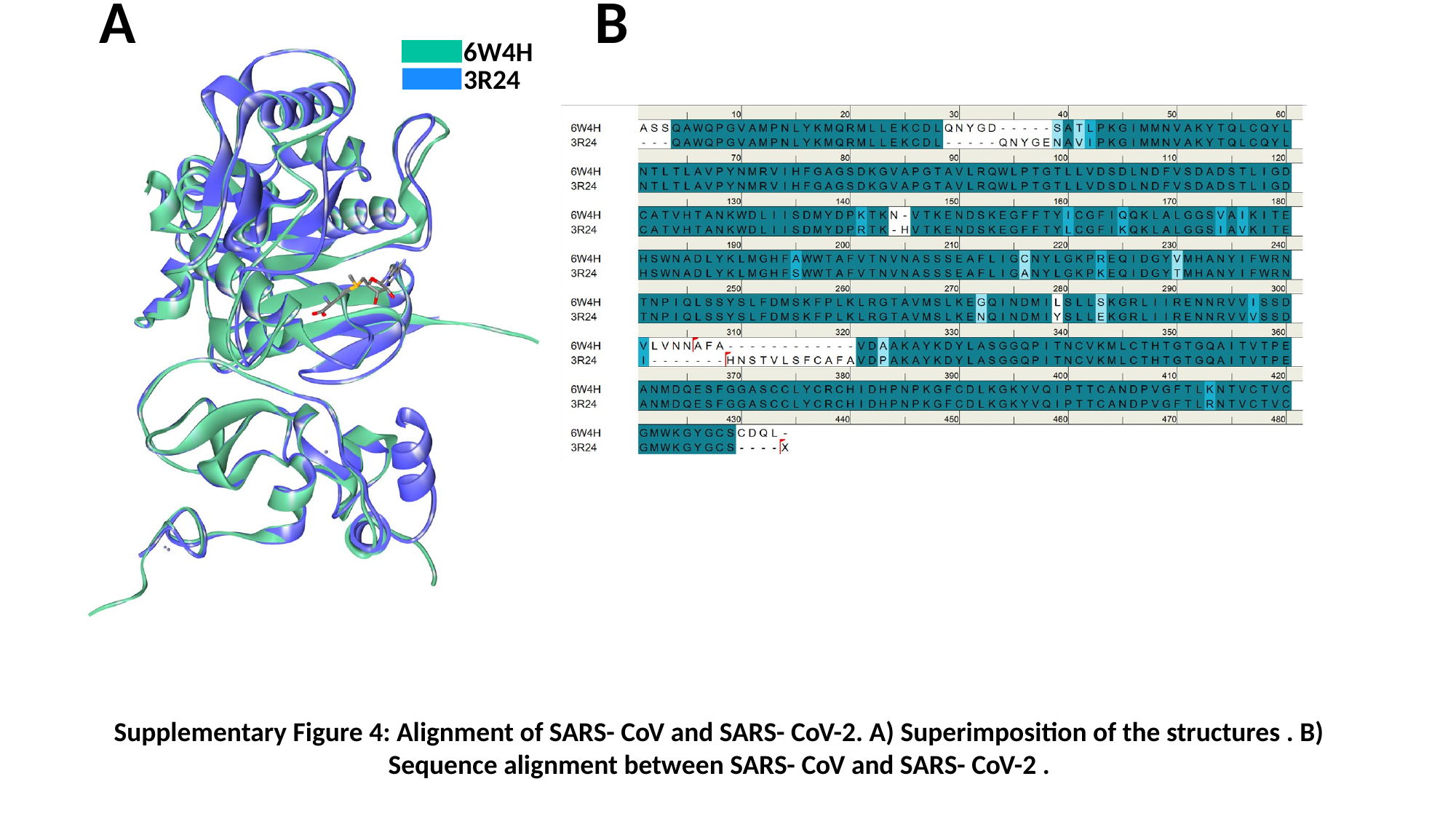

A
B
6W4H
3R24
Supplementary Figure 4: Alignment of SARS- CoV and SARS- CoV-2. A) Superimposition of the structures . B) Sequence alignment between SARS- CoV and SARS- CoV-2 .

## Slide 5
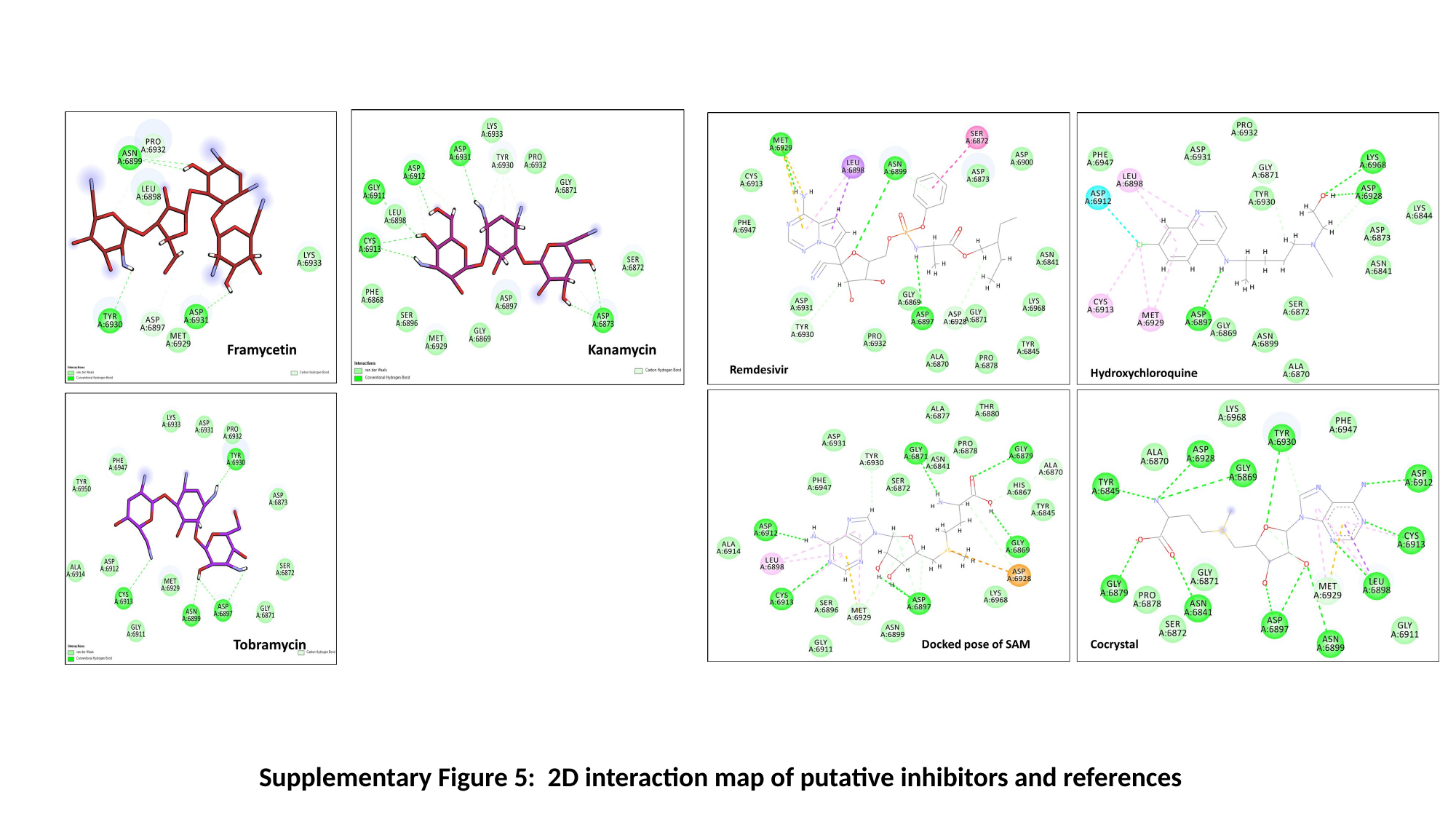

Supplementary Figure 5: 2D interaction map of putative inhibitors and references
